# The wing scales of the mother-of-pearl butterfly, *Protogoniomorpha parhassus*, are thin film reflectors causing strong iridescence and polarization

**DOI:** 10.1242/jeb.242983

**Published:** 2021-08-06

**Authors:** Doekele G. Stavenga

**Affiliations:** Zernike Institute for Advanced Materials, University of Groningen, Nijenborgh 4, 9747AG Groningen, The Netherlands

**Keywords:** Reflectance spectrum, Structural coloration, Wing scales, Color vision, Polarization vision

## Abstract

The dorsal wings of the mother-of-pearl butterfly, *Protogoniomorpha parhassus*, display an angle-dependent pink, structural color. This effect is created by light interference in the lower lamina of the wing scales, which acts as an optical thin film. The scales feature extremely large windows that enhance the scale reflectance, because the upper lamina of ridges and cross-ribs is very sparse. Characteristic for thin film reflectors, the spectral shape of the reflected light strongly depends on the angle of light incidence, shifting from pink to yellow when changing the angles of illumination and observation from normal to skew, and also the degree of polarization strongly varies. The simultaneous spectral and polarization changes serve a possibly widespread, highly effective system among butterflies for intraspecific communication during flight.

## Abstract (precis)

**Summary:** The dorsal wings of the mother-of-pearl butterfly, *Protogoniomorpha parhassus*, show characteristics of thin film reflectors, allowing simultaneous spectral and polarization changes, which may be important in intraspecific communication.

## INTRODUCTION

Natural objects with smooth surfaces often display distinct polarized light patterns, because of the strong dependence of the reflection of light on the angle of incidence and polarization of the illumination. These polarization patterns potentially contain important visual information that is used by many animals, specifically arthropods and cephalopods, that have polarization vision (Marshall et al., 2019). Interestingly, many animals show polarized reflections themselves, as a result of having a smooth skin, hairs or cuticle. These polarized reflections are famously exploited by female tabanids, deerflies and horseflies, which detect their sources for a blood meal by their polarized features (Horváth et al., 2017; Meglič et al., 2019). Moreover, tabanids as well as many beetle species use the polarized light reflections of water surfaces to detect a useful place for positing their eggs (Schwind, 1991; Egri et al., 2012). In the marine environment, cephalopods and crustaceans exploit polarization patterns for predation or communication (Daly et al., 2016; Temple et al., 2021).

A special case of polarization vision concerns the recognition of potential mates by their polarization signal (Marshall et al., 2019). A well-known example is that of *Heliconius* butterflies (Sweeney et al., 2003). When males of the species *Heliconius cydno* and *Heliconius melpomene* were exposed to wings of conspecific females, placed behind filters that either maintained or destroyed the polarized wing reflections, only male *H. cydno* and not *H. melpomene* responded significantly more frequently to female wings with a polarization signal. This finding correlated well with the strongly polarized wing reflections of *H. cydno*, a feature absent in *H. melpomene* (Sweeney et al., 2003).

The polarization pattern of the wings of *H. cydno* butterflies was attributed to iridescent thin films, but their anatomical identity and further optical details were not specified. Polarizing wing reflections of a wide variety of butterflies have also been documented (Douglas et al., 2007), but the optical mechanisms causing the polarization, presumably located in the wing scales, were not discussed. Butterfly wing scales consist of an upper and lower lamina. The upper lamina consists of rows of parallel ridges and cross-ribs, which together frame so-called windows (Ghiradella, 1989, 1998, 2010). The lower lamina is generally a simple, more or less flat, thin plate, that can act as a thin film reflector (Mason, 1927; Stavenga et al., 2014a; Wasik et al., 2014; Giraldo and Stavenga, 2016; Thayer et al., 2020). With a thickness of ∼200 nm, it creates a distinct violet–blue structural color, as is the case in the eye spots of the peacock butterfly *Inachis io* (Stavenga et al., 2014b). This is also the case in certain wing scales of *Heliconius doris*, but in *Heliconius sara* and *Heliconius erato* the blue structural coloration of local wing patches is due to multilayered lamellae in the wing scale ridges (Wilts et al., 2017). The latter method of structural coloration is abundantly practiced by the well-known *Morpho* butterflies (Ghiradella, 1989; Vukusic et al., 1999; Giraldo et al., 2016).

Another outstanding example of structural wing coloration is the forest or common mother-of-pearl butterfly, *Protogoniomorpha parhassus*, a nymphaline butterfly species, also known as *Salamis parhassus*, which belongs to an exclusively Afrotropical genus (Bonte and Van Dyck, 2009). Here, I show that thin film optics determines the color of the wings. Notably, the degree of polarization as well as the color of the reflected light strongly depend on the angle of illumination and view.

## MATERIALS AND METHODS

### Specimens and photography

Specimens of *Protogoniomorpha parhassus* (Drury 1782) were obtained from commercial sources (demuseumwinkel.com). Mounted specimens as well as wing parts were photographed with a Nikon D70 digital camera, equipped with an F Micro-Nikkor lens (60 mm, f2.8; Nikon, Tokyo, Japan). Close-up photographs of small wing areas and isolated scales were made with a Zeiss Universal microscope, using a Zeiss Epiplan 16×/0.35 objective (Zeiss, Oberkochen, Germany).

### Imaging scatterometry

Imaging scatterometry was applied to single scales, glued at the end of pulled glass micropipettes, to visualize the far-field angular distribution of the scattered light. The sample was positioned in the first focal point of the scatterometer's ellipsoidal mirror, which collects light from a full hemisphere. A narrow aperture (5 deg) beam provided by a xenon lamp illuminated a small area of a scale (diameter 13 µm). A piece of magnesium oxide served as a white diffuse reference object. Scatterogram images were acquired by an Olympus DP70 camera (Olympus, Tokyo, Japan; for details, see Stavenga et al., 2009).

### Electron microscopy

Individual scales were removed from the wing and placed onto adhesive carbon tape atop a standard aluminium SEM stub. A 5 nm gold layer was sputtered onto the sample using a 208 HR sputter coater (Cressington Scientific Instruments, Watford, UK) to prevent charging. The sample was subsequently imaged using a Scios 2 dual-beam field emission electron microscope (FEI, Eindhoven, The Netherlands).

### Spectrophotometry

Reflectance spectra of single scales and scale-less wing areas were measured with a microspectrophotometer (MSP), consisting of a Leitz Ortholux microscope with a LUCPlanFL N 20×/0.45 objective (Olympus) and an Avantes AvaSpec-2048-2 CCD detector array spectrometer (Avantes, Apeldoorn, The Netherlands), with a xenon lamp light source. The reference was a white diffuse standard (Avantes WS-2). Wing reflectance spectra were measured with an integrating sphere and a bifurcated probe connected to a halogen/deuterium light source and the Avantes spectrometer. Reflectance spectra of the intact wing were also measured as a function of angle of light incidence for both transverse electric (TE)- and transverse magnetic (TM)-polarized light (where light is polarized perpendicular and parallel to the plane of light incidence, respectively) in a goniometric setup with two rotatable optical fibers. One fiber delivered light from a xenon lamp to the object, and the other fiber collected the reflected light and guided it to the spectrometer. The angular resolution of the setup has a Gaussian shape with half-width ∼5 deg (Stavenga et al., 2011). Naturally, the measured spectra slightly varied in shape and magnitude. The data in Figs 3–5 are representative, single cases.

### Modeling thin film reflectance spectra

The reflectance spectra of chitinous thin films were calculated as a function of the angle of light incidence using the classical Airy formulae (Yeh, 2005; Stavenga, 2014; Stavenga et al., 2018) and the wavelength-dependent refractive index of butterfly chitin (Leertouwer et al., 2011) for both TE- and TM-polarized light.

### Calculating photoreceptor signals

How the butterflies' wing reflections will be detected by conspecifics can be assessed by assuming a set of three classes of photoreceptors with maximal sensitivity in the ultraviolet (UV), blue (B) and green (G) wavelength ranges encountered in some related nymphalids (Kinoshita et al., 1997). The relative signals created in the receptor classes *i*=1–3 are:(1)
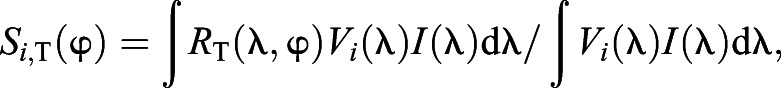
where *R*_T_(λ,ϕ) is the reflectance spectrum as a function of wavelength λ and angle of light incidence ϕ for either *T*=TE- or TM-polarized light; *V_i_*(λ) is the absorption spectrum of the visual pigment of receptor class *i* calculated with peak wavelength 350 nm (UV), 440 nm (B) and 530 nm (G), respectively, applying standard formulae (Govardovskii et al., 2000; Stavenga, 2010); and *I*(λ) is the solar spectrum converted into a photon flux and normalized (derived from https://www.pveducation.org/pvcdrom/appendices/standard-solar-spectra). For unpolarized light, the angle-dependent receptor signal is:(2)
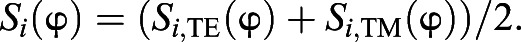
The degree of polarization of the signals created by TE- and TM-polarized light:(3)
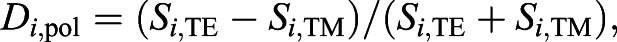
was calculated for both the wing reflectance and an ideal thin film with thickness 160 nm.

## RESULTS

### Scales on the dorsal wing side

**Fig. 1.**
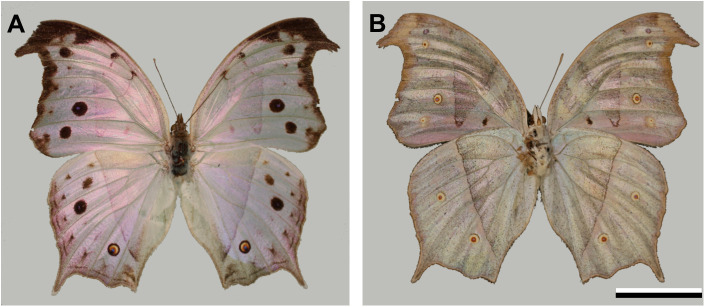
The mother of pearl **butterfly**, *Protogoniomorpha parhassus*. (A) Dorsal wing side. (B) Ventral wing side. Scale bar: 2 cm.

The dorsal sides of both the fore- and hind-wings of *P. parhassus* display a marked opalescent pink sheen, dotted with some dark spots, together with a brown-black margin (Fig. 1A). The ventral wing sides have an overall pale brownish pattern with a rosy-pink tinge (Fig. 1B).

**Fig. 2.**
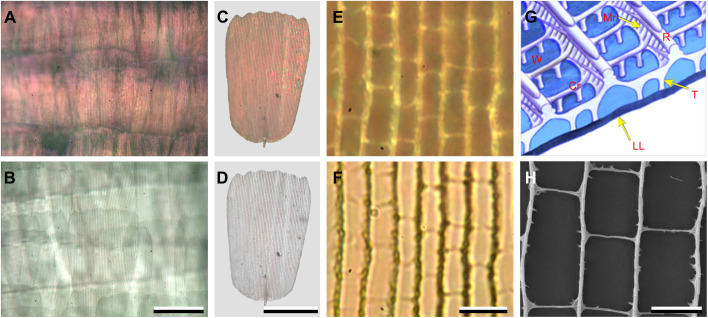
**Wing scales of the dorsal wings of *P. parhassus*.** (A) Epi-illumination of the dorsal forewing. (B) The same region of the forewing examined with transmitted light. (C) Epi-illumination of an isolated scale in air on a microscope slide. (D) The same scale in transmitted light. (E) Close-up view; epi-illumination. (F) Close-up view; transmitted light. (G) Diagram of a basic nymphalid wing scale (adapted from Wasik et al., 2014). R, ridge; Mr, microrib; Cr, cross-rib; T, trabecula; LL, lower lamina; W, window. (H) FIB-SEM scanning electron micrograph. Scale bars: A–D, 100 µm; E,F, 10 µm; H, 3 µm.

As the origin of this coloration must be the wing scales, I examined an intact forewing with a light microscope, applying both epi-illumination and transmitted light (Fig. 2A,B). The pink color seen with epi-illumination of the dorsal forewing vanished in transmitted light, which demonstrates the structural origin of the colors. Observation of an isolated forewing scale on a microscope slide underscores this conclusion, as epi-illumination of the scale shows the pink color, while with transmitted light the scale is colorless (Fig. 2C,D). Inspecting the scale at high magnification shows the classical organization of nymphaline wing scales, with regularly arranged parallel ridges and somewhat irregularly spaced cross-ribs (Fig. 2E,F). Upon illumination, the ridges, which consist of overlapping lamellae and microribs, as well as the cross-ribs and the trabeculae that connect the upper and lower laminae (Fig. 2G), all act as scatterers and thus become bright in epi-illumination (Fig. 2E) and dark in transmitted light (Fig. 2F). Strikingly, whereas in the wing scales of other nymphalines the distance between adjacent ridges and cross-ribs is typically 1–2 µm (Stavenga et al., 2014b), here the ridges and cross-ribs with distances >3 µm are very widely spaced, thus creating extremely large windows (Fig. 2G). Nearly the full lower lamina is thus exposed to incident light (Fig. 2E,F,H).

**Fig. 3.**
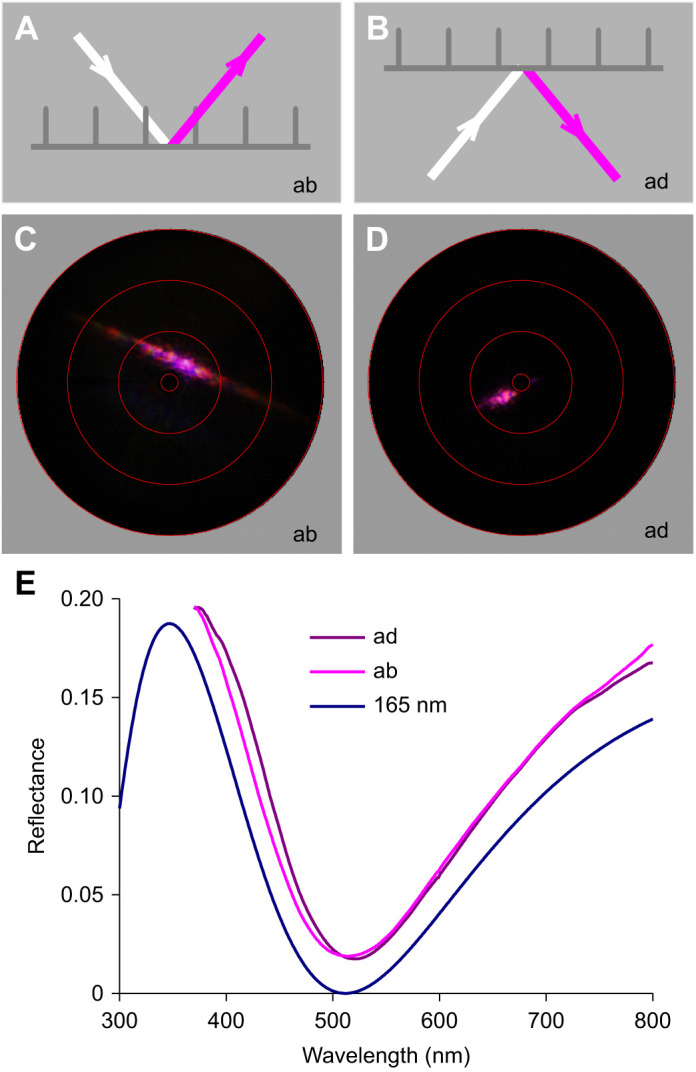
**Scatterometry and microspectrophotometry on single wing scales of *P. parhassus*.** (A) Diagram of abwing (the upper side of the scale) illumination of a wing scale. (B) Diagram of adwing (the scale side facing the wing) illumination. (C) Abwing scatterogram. (D) Adwing scatterogram. The circles in C and D represent angular directions of 5, 30, 60 and 90 deg. (E) Reflectance spectra measured with a microspectrophotometer (MSP) from the adwing and abwing sides of the scales compared with the spectrum of an ideal chitinous thin film with 165 nm thickness.

The lower laminae of lepidopteran wing scales generally act as dielectric thin films, and presumably therefore the pink color of *P. parhassus* is also due to thin film reflections of the dorsal wing scales, as conjectured nearly 100 years ago in the only published report on *P. parhassus* coloration (Onslow, 1923). I therefore investigated the reflection properties of isolated, single scales, applying imaging scatterometry and microspectrophotometry (Fig. 3). A narrow aperture beam of white light focused onto a small area on the abwing (upper) side of an isolated dorsal wing scale created a pinkish diffraction pattern (Fig. 3A,C). The direction of the line pattern in the scatterogram is perpendicular to the ridge grating. The scatterogram of the adwing (under) side showed only a very local, similar pinkish-colored spot (Fig. 3B,D). The two patterns can be immediately understood to result from the scale's structure, where the regularly spaced ridges will act as a grating with a period of ∼3 µm (Fig. 3A) and the lower lamina is a thin film reflector (Fig. 3B).

Microspectrophotometry of both scale sides (Fig. 3E) revealed spectra that closely resembled the reflectance spectrum calculated for a normally illuminated chitinous thin film with 165 nm thickness (Fig. 3E). With a white diffuser as reference, their amplitude is overestimated, but when corrected with a factor 0.5 (abwing, Fig. 3E) and 0.2 (adwing, Fig. 3E), the spectra were virtually identical to the calculated spectrum, except for a minor offset (Fig. 3E). The latter must be attributed to scattering by the ridges and cross-ribs of the upper lamina as well as to the large numerical aperture objective used in the MSP, so that the reflectance is an integral of slightly varying, angle-dependent spectra (see Stavenga, 2014).

**Fig. 4.**
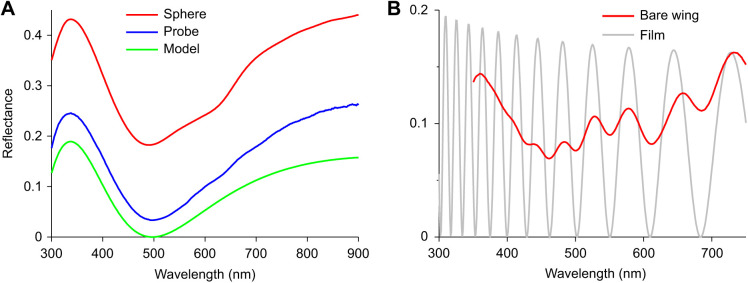
**Reflectance spectra of the dorsal forewing of *P. parhassus*.** (A) Spectra measured with an integrating sphere and a bifurcated reflection probe, compared with the reflectance spectrum calculated for a chitinous thin film with thickness 160 nm. (B) Reflectance spectrum of a bare wing substrate measured with a MSP, compared with the reflectance spectrum calculated for a thin film with thickness 1.78 µm.

The spectra measured with the MSP were limited to wavelengths above 350 nm and furthermore suffered from an uncertainty in the absolute reflectance value. I therefore performed measurements with an integrating sphere, which integrates the reflectance over all scattering angles, similar to the case for the white diffuser reference. This yielded a spectrum that can be quantitatively related to that of an ideal chitinous thin film. In fact, except for a considerable offset, the spectrum (Fig. 4A, sphere) was virtually identical to that of a thin film with thickness 160 nm, which has a distinct peak in the UV wavelength range, at 338 nm, and a minimum at 498 nm (Fig. 4A, model).

The additional offset can be understood to originate from the material beneath the scales, i.e. the wing substrate and the scales at the ventral wing side, particularly as the dorsal wing scales reflect only a minor part of the incident light. The scales form approximately a uniform layer with little overlap, so that with about normal illumination the reflectance of the scale layer will be no more than ∼0.2. In other words, at least 80% of the incident light will reach the underlying wing substrate. To assess the wing substrate's contribution to the measured reflectance, I measured the wing reflectance spectrum with a MSP at local areas devoid of scales (Fig. 4B). The measured spectrum showed oscillations characteristic of a rough thin film with mean thickness 1.78 µm (Fig. 4B) and an average reflectance of the order of 10%. The fraction of the incident light reflected by the intact wing hence will be ∼0.1×0.8=0.08. At least 80% of this fraction will be transmitted by the scales again, thus contributing to the total reflection a background signal of about 0.8×0.1×0.8, or ∼6%. Moreover, the light transmitted by the wing substrate will reach the scales at the ventral wing sides and be partly reflected and scattered there. Together, these events will result in a wide aperture beam of reflected light, which thus can contribute a considerable background offset to the total wing reflectance, as was measured with the integrating sphere (Fig. 4A).

A reflectance spectrum similar to that obtained with the sphere was measured with a bifurcated reflection probe, which collects the reflection from a 1–2 mm sized light spot. As the white reference is a diffuser and the wing, instead, is a directional reflector, an estimated correction factor of 0.3 was applied, which yielded the spectrum shown in Fig. 4A. The background is here much smaller than with the sphere, because the probe collects only light from within a small aperture.

### Angle-dependent reflections and polarization

**Fig. 5.**
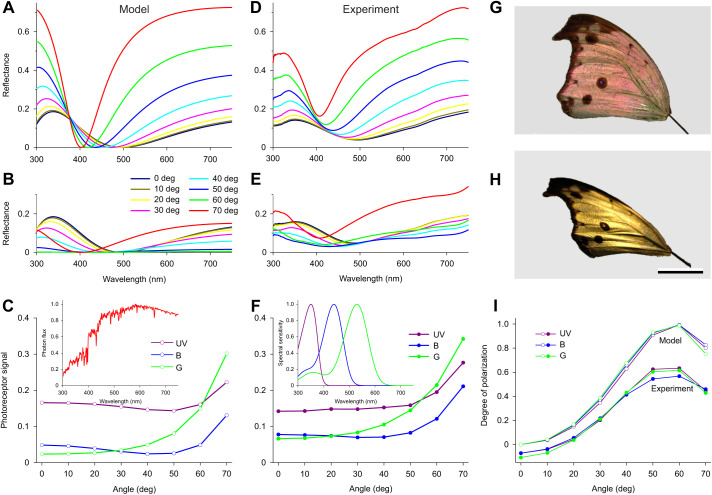
**Angle-dependent reflectance spectra and degree of polarization.** (A) TE-reflectance spectra of a thin film of thickness 160 nm as a function of the angle of light incidence. (B) The corresponding TM-reflectance spectra. (C) Signals generated in UV, B and G photoreceptors (F inset, spectral sensitivities) by unpolarized sunlight (inset, normalized spectrum) illuminating the model thin film of A and B as a function of angle of light incidence. (D) Reflectance spectra of an intact forewing of *P. parhassus* for TE-polarized incident light. (E) Reflectance spectra of the forewing for TM-polarized light. (F) Signals generated in UV, B and G photoreceptors (inset, spectral sensitivities) by unpolarized sunlight (C inset, normalized spectrum) illuminating the forewing of D and E as a function of angle of light incidence. (G) A dorsal forewing under normal illumination and view. (H) The dorsal forewing illuminated with unpolarized light at angle of incidence of 50 deg and viewed from the mirror angle; scale bar: 1 cm. (I) Angle dependence of the degree of polarization derived from A and B (model) and D and E (experiment).

A well-known characteristic of thin films is the strong spectral and polarization dependence of the reflectance spectra on the angle of light incidence. Fig. 5A,B shows the reflectance spectra of a thin film with thickness 160 nm. The spectra show a distinct hypsochromic (toward shorter wavelengths) spectral shift with an increasing incidence angle. The reflectance minimum changes from 500 nm with normal illumination to 400 nm at a skew illumination of 70 deg. Furthermore, whereas the reflectance amplitude of TE-polarized light rises with increasing angle of incidence (Fig. 5A), the reflectance amplitude of TM-polarized light diminishes, becoming zero at a Brewster's angle of ∼60 deg (Fig. 5B; Brewster's angle for chitin is 57 deg at 500 nm; Leertouwer et al., 2011).

To investigate whether the butterfly wings feature the same characteristics as a thin film, I measured the angle-dependent reflectance spectra of the dorsal forewing. I applied a narrow-aperture light beam at various angles of incidence with an optical fiber and then measured the reflected light flux into the mirror angle with another optical fiber for both TE- and TM-polarized light (Fig. 5A,B). The plane of light incidence was about parallel to the longitudinal axis of the scale ridges. As expected, as with the ideal 160 nm thin film, both the shape and peak wavelength of the reflectance spectra strongly changed when increasing the angle of light incidence (Fig. 5D,E).

The changes in the reflectance spectra occurring when the direction of illumination changes will affect the wing color of a flying butterfly seen by other butterflies. Let us consider the 160 nm thin film illuminated by unpolarized sunlight (as that of the inset in Fig. 5C) that is observed by a nymphalid butterfly with trichromatic color vision that is served by photoreceptors with visual pigments absorbing maximally at 350, 440 and 530 nm, i.e. with UV, B and G photoreceptors having spectral sensitivities as shown in the inset of Fig. 5F (e.g. Kinoshita et al., 1997). The signals (calculated with Eqn 2) then created by the ideal thin film in the UV and B receptors appear to be hardly angle dependent, but the signal in the G receptor steeply increases when the angle of incidence becomes larger than ∼50 deg (Fig. 5C). The dorsal wing of *P. parhassus* shows a very similar behavior (Fig. 5F), and thus a wing flapping butterfly will show a strongly varying color, as illustrated in Fig. 5G,H.

The degree of polarization of the receptor signals, calculated with Eqn 3, also changes greatly with increasing angle of illumination, but the degree of change hardly differs between the three receptor classes (Fig. 5I). Of course, the angle dependence is more pronounced for the ideal thin film (Fig. 5I, model), but the dorsal wing of *P. parhassus* also demonstrates a strongly angle-dependent degree of polarization (Fig. 5I, experiment). Clearly, both color and polarization of the wing reflections strongly vary with the angle of illumination, which thus presumably create highly effective, dynamic recognition signals.

### Scales on the ventral wing side

**Fig. 6.**
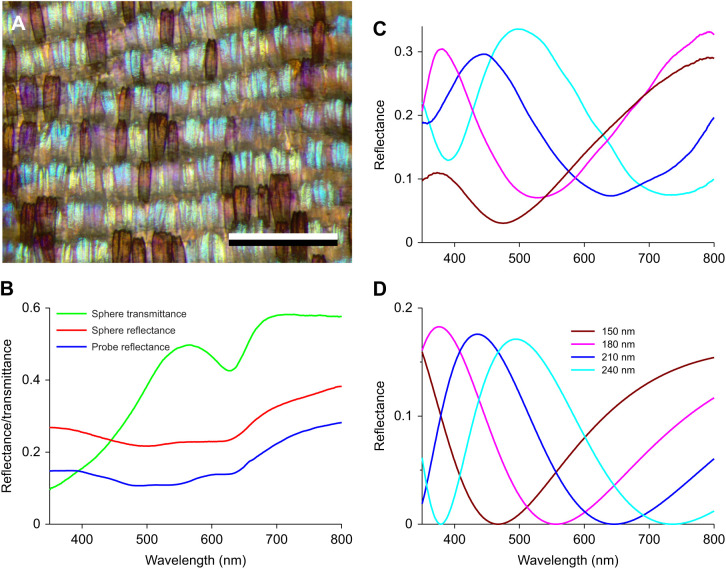
**Spectral characteristics of the ventral wing.** (A) The multicolored lattice of scales at the ventral wing, observed with epi-illumination; scale bar: 0.5 mm. (B) Reflectance and transmittance spectra measured with an integrating sphere and bifurcated probe with illumination incident on the ventral forewing. (C) Reflectance spectra measured with a MSP of individual ventral wing scales (line color similar to the scale color). (D) Reflectance spectra of chitinous thin films with thickness 150, 180, 210 and 240 nm.

Whereas the scales at the dorsal wing side virtually uniformly show the same pink color, the scales on the ventral wing vary extensively, displaying purple, blue, yellow or brown colors (Fig. 6A). Reflectance spectra of the ventral forewing measured with the integrating sphere and the bifurcated probe thus yielded only slightly varying spectra, because they represent the cumulative reflectance of several scales (Fig. 6B). However, reflectance spectra measured from individual scales with an MSP strongly differed from each other (Fig. 6C). Their broad-band shape resembled that of the dorsal wing scales, which suggests that the lower lamina of the ventral scales also acts as a thin film reflector and thus determines the color of the scales. Presumably, the difference in the spectral location of the reflectance band was due to a varying thickness of the lower laminae. This hypothesis is exemplified by Fig. 6D, which shows the reflectance spectra of chitinous thin films with thickness varying between 150 and 240 nm.

As brown-colored butterfly wing scales usually contain melanin, I performed transmittance measurements on the complete wing with the integrating sphere, which yielded an increasing transmittance with increasing wavelength, as is indeed the case with melanin (Fig. 6B). Curiously, however, the transmittance spectra had a distinct valley with a minimum at 630 nm (Fig. 6B), indicating the presence of a pigment different from melanin. The unknown pigment is located in the wing substrate, as very similar spectra were obtained when measuring the transmittance of wing areas where scales were removed with the MSP.

## DISCUSSION

The wings of Lepidoptera generally display striking colors, which have either a pigmentary or a structural origin, and often the two coloration mechanisms are combined to reinforce the coloration pattern. Nymphalid butterflies are generally colored by ommochromes and their precursor 3-OH-kynurenine, but also by the ubiquitous melanin (Koch, 1993; Zhang et al., 2017). The wings of the mother-of-pearl butterfly, *P. parhassus*, are certainly colorful, but this is realized by the scales acting as thin film reflectors, which is accentuated by a few black spots and black wing margins. Still, I did encounter a deviant pigment in the wing substrate, so far unknown, with an absorption band peaking at 630 nm. The distinctly absorbing wing substrate of the papilionid *Graphium sarpedon* contains the bile pigment sarpedobilin, which has a clear absorption peak at 670 nm, together with the blue-absorbing carotenoid lutein (Stavenga et al., 2010). In *G. sarpedon*, the pigments play a distinct role in wing coloration. Another uncharacterized pigment, peaking at 720 nm, was found in the wings of some *Heliconius* species (Wilts et al., 2017). Possibly the unknown wing pigments are various bile pigments or tetrapyrroles, but their function remains presently obscure.

The structural coloration of lepidopteran wings has been the subject of numerous studies, and the various underlying structures have been well classified into seven categories (Ghiradella, 1998). Somewhat surprisingly, Ghiradella's classification did not include the lower scale lamina acting as a thin film reflector. This was quite appropriately noticed by Kinoshita (2008), who added it as the eighth category, but its function is of course much broader than only an optical diffuser, as found in the case of a cover scale in *Morpho didius* (Kinoshita, 2008). For instance, in many cases the lower lamina acts as a thin film blue reflector, as in the peacock butterfly *I. io* (Stavenga et al., 2014b; Wilts et al., 2017). A somewhat more sophisticated case is that of the green scales of *H. doris*, where 3-OH-kynurenine acts as a short-wavelength filter in front of a blue-reflecting lower lamina (Wilts et al., 2017). Perhaps an even more important function is realized in several cases of pigmented scales where the reflectance spectrum of the lower lamina is tuned to that of the pigmented upper lamina, so enhancing the coloration. The thickness of the lower lamina is then carefully adjusted to the absorption properties of the scale's pigment (Stavenga et al., 2014b, 2015; Thayer et al., 2020).

The thin film reflections of the lower lamina are essentially unavoidable, yet there are many cases where they are suppressed. Most butterflies create black scales by expressing melanin in the upper lamina. The melanin thus effectively blocks the lower lamina twofold, as only a small fraction of incident light reaches the lower lamina and even less of the light reflected by the lower lamina then passes the upper lamina on the way back (e.g. Stavenga et al., 2014b). The scales become brown with moderate amounts of melanin, which is the case in most moths (Stavenga et al., 2020). Yet, Mason (1927) noticed that a primitive moth, a washed purple *Eriocrania* sp., had scales colored by their basal membrane that ‘behaves as if it were a single thin film’, but he found that the reflection color (purplish to orange) was relatively faint. Since then, the crucial role of the lower lamina for the coloration of lepidopterans had become largely forgotten, but it is recently becoming more recognized (Trzeciak et al., 2012; Wasik et al., 2014; Stavenga et al., 2014b, 2018; Siddique et al., 2016; Thayer et al., 2020).

*Protogoniomorpha parhassus* appears to be special in that virtually the full dorsal wings are studded with scales that are colored as a result of the thin film properties of their lower lamina. With normal illumination, the reflectance is certainly rather weak, of the order of 10%, but with an increasing angle of illumination, the wing gains substantially in reflectance and shifts in coloration (Fig. 5F). Most crucially, the degree of polarization concomitantly changes strongly, with a peak at the Brewster angle of the thin film reflectors (Fig. 5I).

The simultaneous changes in color and polarization will create a detection problem for the butterflies (Kelber et al., 2001; Kinoshita and Arikawa, 2014). The discrimination of color unconfounded by polarization requires polarization-insensitive receptors, which can be achieved by twisting the rhabdomeres (Wehner and Bernard, 1993). Reliable polarization vision requires a set of differently arranged polarization sensors with the same spectral sensitivity (Bernard and Wehner, 1977). Such a system is realized in the dorsal rim area of many insects (Labhart, 2016), but the organization of similar systems in the main retina and their signal processing is far from clarified. Nevertheless, as shown in Fig. 5I, all photoreceptor classes assumed to act in *P. parhassus* can perform this task well, but given the usual majority of green receptors and the high long-wavelength reflectance, the green receptors will be the most suitable for this task, especially as the habitat of the butterflies is open forest, riverine bush, savanna and forest margins (see https://www.metamorphosis.org.za/articlesPDF/1145/153%20Genus%20Protogoniomorpha%20Wallengren.pdf).

In conclusion, the mother-of-pearl butterfly, *P. parhassus*, by having wings with a scale layer functioning as thin reflectors, possesses a potentially unique signaling system that creates strong changes of color as well as polarization during flight, i.e. when the angular position of the wings rapidly changes. Male *H. cydno* can discriminate the conspecific females via polarized reflections of static displayed wings (Sweeney et al., 2003). Presumably, flying wings will exert even stronger, dynamic polarized signaling. As polarizing wings are widespread among butterflies (Douglas et al., 2007), it will be very interesting to investigate the role of color versus polarization in angle-dependent wing signaling for interspecific communication in *P. parhassus* and other butterfly species.
